# Evaluation of gantry speed on image quality and imaging dose for 4D cone-beam CT acquisition

**DOI:** 10.1186/s13014-016-0677-8

**Published:** 2016-07-29

**Authors:** Andrew P. Santoso, Kwang H. Song, Yujiao Qin, Stephen J. Gardner, Chang Liu, Indrin J. Chetty, Benjamin Movsas, Munther Ajlouni, Ning Wen

**Affiliations:** 1 Department of Radiation Oncology, Wayne State University School of Medicine, Detroit, MI 48201 USA; 2 Texas Oncology, Fort Worth, TX 76104 USA; 3 Department of Radiation Oncology, Henry Ford Health System, Detroit, MI 48202 USA

**Keywords:** Cone-beam CT, Image guidance, Dosimetry, Motion management

## Abstract

**Background:**

This study investigates the effect of gantry speed on 4DCBCT image quality and dose for the Varian On-Board Imager®.

**Methods:**

A thoracic 4DCBCT protocol was designed using a 125 kVp spectrum. Image quality parameters were evaluated for 4DCBCT acquisition using Catphan® phantom with real-time position management™ system for gantry speeds varying between 1.0 to 6.0°/s. Superior-inferior motion of the phantom was executed using a sinusoidal waveform with five second period. Scans were retrospectively sorted into 4 phases (CBCT-4 ph) and 10 phases (CBCT-10 ph); average 4DCBCT (CBCT-ave), using all image data from the 4DCBCT acquisitions was also evaluated. The 4DCBCT images were evaluated using the following image quality metrics: spatial resolution, contrast-to-noise ratio (CNR), and uniformity index (UI). Additionally, Hounsfield unit (HU) sensitivity compared to a baseline CBCT and percent differences and RMS errors (RMSE) of excursion were also determined. Imaging dose was evaluated using an IBA CC13 ion chamber placed within CIRS Thorax phantom using the same sinusoidal motion and image acquisition settings as mentioned above.

**Results:**

Spatial resolution decreased linearly from 5.93 to 3.82 lp/cm as gantry speed increased from 1.0 to 6.0°/s. CNR decreased linearly from 4.80 to 1.82 with gantry speed increasing from 1.0 to 6.0°/s, respectively. No noteworthy variations in UI, HU sensitivity, or excursion metrics were observed with changes in gantry speed. Ion chamber dose rates measured ranged from 2.30 (lung) to 5.18 (bone) E-3 cGy/mAs.

**Conclusions:**

A quantitative analysis of the Varian OBI’s 4DCBCT capabilities was explored. Changing gantry speed changes the number of projections used for reconstruction, affecting both image quality and imaging dose if x-ray tube current is held constant. From the results of this study, a gantry speed between 2 and 3°/s was optimal when considering image quality, dose, and reconstruction time. The future of 4DCBCT clinical utility relies on further investigation of image acquisition and reconstruction optimization.

## Background

Stereotactic body radiotherapy (SBRT) has become a form of treating inoperable non-small cell lung cancer (NSCLC) in its early stages [1]. Work conducted by Onishi et al. showed that patients staged with T1 and T2 NSCLC treated with SBRT had cumulative local control rates better than 70 % at 5 years; this is in contrast to local control rates of 50 % with a 5-year survival of approximately 15–30 % for patients treated with conventionally fractionated radiotherapy [1]. Five-year relative survival rates for lung cancers remains somewhat low at 18 %, attributed to more than one-half of diagnoses made at a distant stage [2].

Though initial clinical results of SBRT for lung cancer are promising, there remain technical complexities that must be addressed. In particular, localization error associated with the treatment of moving targets in lung SBRT must be minimized [3]. The ability of four-dimensional CT (4DCT) to map motion and tissue deformation during respiration, while reducing artifacts, allows for accurate targeting of tumors in the thorax [4, 5]. Strategies to improve target coverage, such as breath hold treatments, gated delivery, and mid-position treatments can also be utilized as part of an approach for respiratory motion management [4, 6, 7]. Margin reduction is also possible for SBRT, having subsequent implications for reducing mean lung dose [8].

Interventional imaging techniques such as cone-beam CT (CBCT) have become clinical standards in image-guided radiotherapy for soft tissue-based target localization and positioning [9]. Linear accelerators can be mounted with a kV source and flat panel detector, allowing for volumetric image acquisition. These volumetric images allow for accurate soft tissue localization and retrospective dose calculation [10]. However, large magnitudes of intra-scan motion in the reconstructed image can lead to clinically dosimetric discrepancies due to poor image quality.

Tumor motion at the treatment position can be assessed using 4DCBCT [11]. Conventional free-breathing CBCT tends to underestimate the tumor extent (i.e., the internal target volume) by as much as 24.2 to 40.1 % depending on tumor size and interfraction variability [3, 12, 13]. To allow for binning of projection images, a surrogate respiratory signal is used to determine amplitude, phase, or temporal information; various methods have been utilized for the respiratory cycle surrogate, including tracking the motion of the diaphragm, mapping changes of the skin surface, thoracic transducer belts, or infrared reflective markers [14]. The 4D reconstruction involves a retrospective correlation of the timing of projection images to the breathing cycle surrogate parameter of interest (i.e., phase or amplitude of respiratory cycle) [4, 15].

The clinical utility of 4DCBCT is dependent on both image quality and imaging dose. Dosimetric studies have been conducted for patient skin dose using thermoluminescent dosimeters as well as Gafchromic film inserted into homogeneous phantoms for different CBCT protocols [16, 17]. Wen et al. reported cumulative dose levels to the left femoral head from daily kV CBCT of pelvic sites can be upwards of 400 cGy. Since 4DCBCT has great potential in localizing lesions in the thorax, it would be worthwhile to understand the dose delivered from 4DCBCT in a heterogeneous medium and the effect of changing practical variables in 4D protocols such as gantry speed due to gantry speed’s inverse relationship with the number of projections. This study investigates the effect of gantry speed on 4DCBCT image quality and dose using the On-Board Imager® (OBI) on the Edge™ radiosurgery system (Varian Medical System, Palo Alto, CA).

## Materials and methods

### CBCT parameterization

All images were acquired with 125 kVp x-ray tube setting. Phantoms were placed at isocenter and were imaged using a half-fan field of view with a half-bowtie filter and full trajectory slightly greater than 360° acquisition, reflecting a typical thoracic region protocol in which a larger field of view is required to prevent truncation of anatomy (see Table 1). A relatively large focal spot of 1.0 mm was used to mediate heat loss. The source-to-detector distance was 150 cm. These fixed parameters mirrored a standard thoracic CBCT clinical protocol.

**Table 1:** Technique factors and parameters set with a variable gantry speed. All images were acquired with 125 kVp x-ray tube setting

Gantry speed (*°*/s)	Field of view (cm)	Matrix size	Pixel size (mm)	Slice thickness (mm)	Tube voltage (kVp)	mAs	Projections	Δ*t* (min)
1.0	46.5	512 × 512	0.7	2.0	125	5716	5400	6.0
2.0	46.5	512 × 512	0.7	2.0	125	2856	2700	3.0
3.0	46.5	512 × 512	0.7	2.0	125	1903	1800	2.0
4.0	46.5	512 × 512	0.7	2.0	125	1427	1350	1.5
5.0	46.5	512 × 512	0.7	2.0	125	1140	1080	1.2
6.0	46.5	512 × 512	0.7	2.0	125	949	900	1.0

**Fig. 1:**
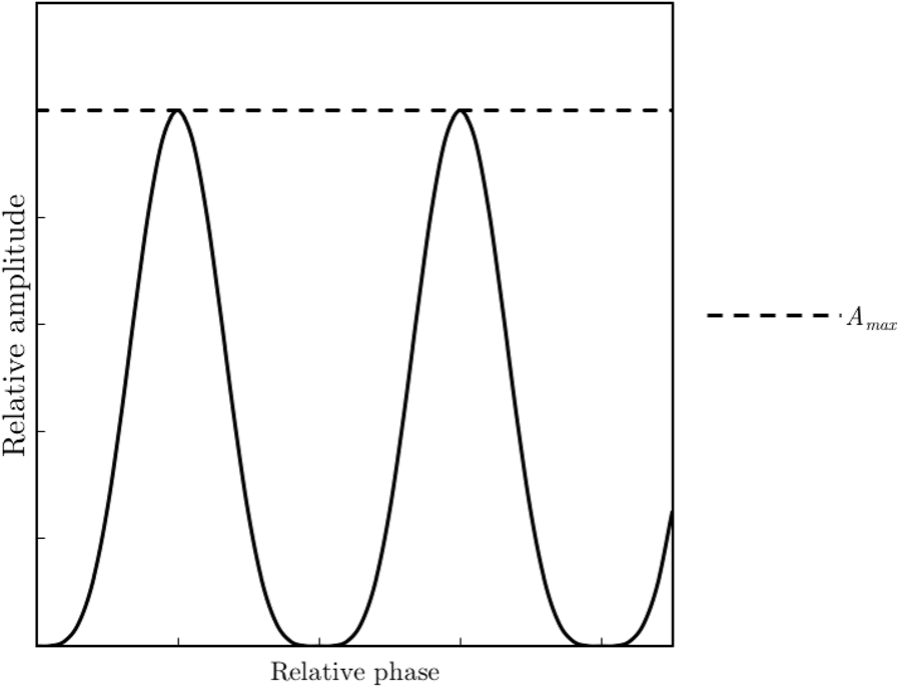
Idealized surrogate sinusoidal respiratory signal mapped as relative amplitude as a function of relative phase. The signal is discretized into a number of phase bins determined by the user allowing for retrospective 4D reconstruction

**Fig. 2:**
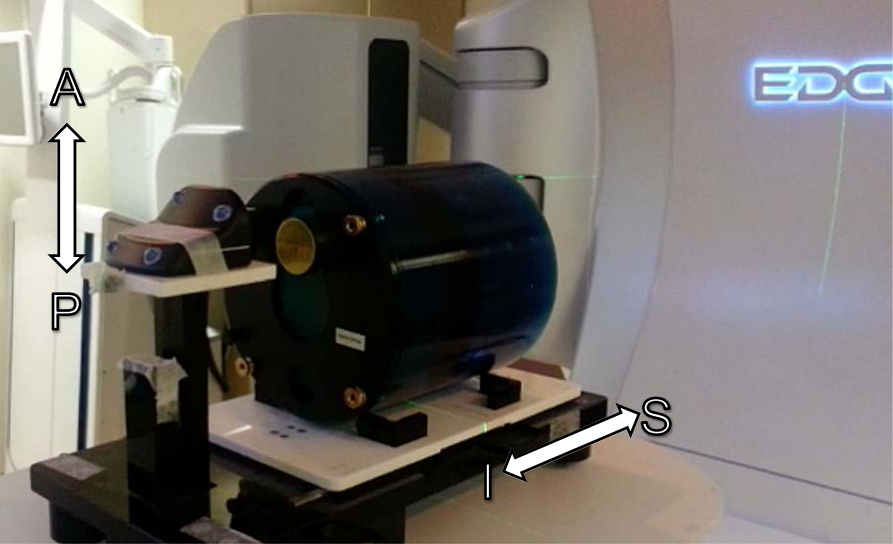
Equipment used in 4D analysis. The Catphan sits on the SI platform while the block sits on the AP platform. The motion of the block is recorded and used as the surrogate signal in 4D reconstruction

The standard Feldkamp-Davis-Kress (FDK) reconstruction algorithm was used in producing average (CBCT-ave) and 4D reconstructions for all acquisitions [18]. Projections were sorted retrospectively into bins according to their respiratory phase corresponding to a surrogate signal, as seen in Fig. 1. The surrogate signal was generated by placing an infrared reflective marker block on a moving anterior-posterior (AP) platform of a BrainLAB ExacTrac gating system phantom (BrainLAB, Heimstetten, Germany) used in tandem with the real-time position management (RPM) system™ (Varian Medical Systems, Palo Alto, CA) to track the motion of the block (see Fig. 2) [19]. An independent computer controlled the motion of the gating phantom’s platforms. A well-defined sinusoidal waveform simulated regular breathing cycles, representing an idealized model for tumor motion [20–22]. The amplitude of waveform motion *A* in cm as a function of phase *ϕ* is given by:

1 $$ A={A}_o{\left(1- \cos \left(2\pi \phi \right)\right)}^2;\phi \in \left[0,1\right] $$

where *A*_0_ represents an amplitude constant in cm. The RPM system™ tracks the block’s motion with an infrared camera and assigns projections to appropriate phase bins used for retrospective reconstruction. The Catphan® 504 phantom was used to evaluate CBCT image quality (Phantom Laboratory, Salem, NY) by placing it on a lower moving superior-inferior (SI) platform of the gating phantom that moved in phase with the infrared marker block’s platform.

An array of parameters can be varied for CBCT acquisition. The number of projections acquired directly relates to image quality and imaging dose [23]. The number of projections *N* for a given CBCT protocol is determined by:

2 $$ N = F\Delta t=F\left(\theta /\omega \right) $$

where *F* is the frame rate and Δ*t* is the acquisition time determined by the angular displacement *θ* and inversely by the gantry speed *ω*. Thus, *N* varies inversely with *ω*. Six protocols were designed by varying gantry speed in integer steps from 1.0 to 6.0° per second (°/s) at a fixed frame rate of 15 frames per second (fps) (see Table 1).

### Image quality analysis

**Fig. 3:**
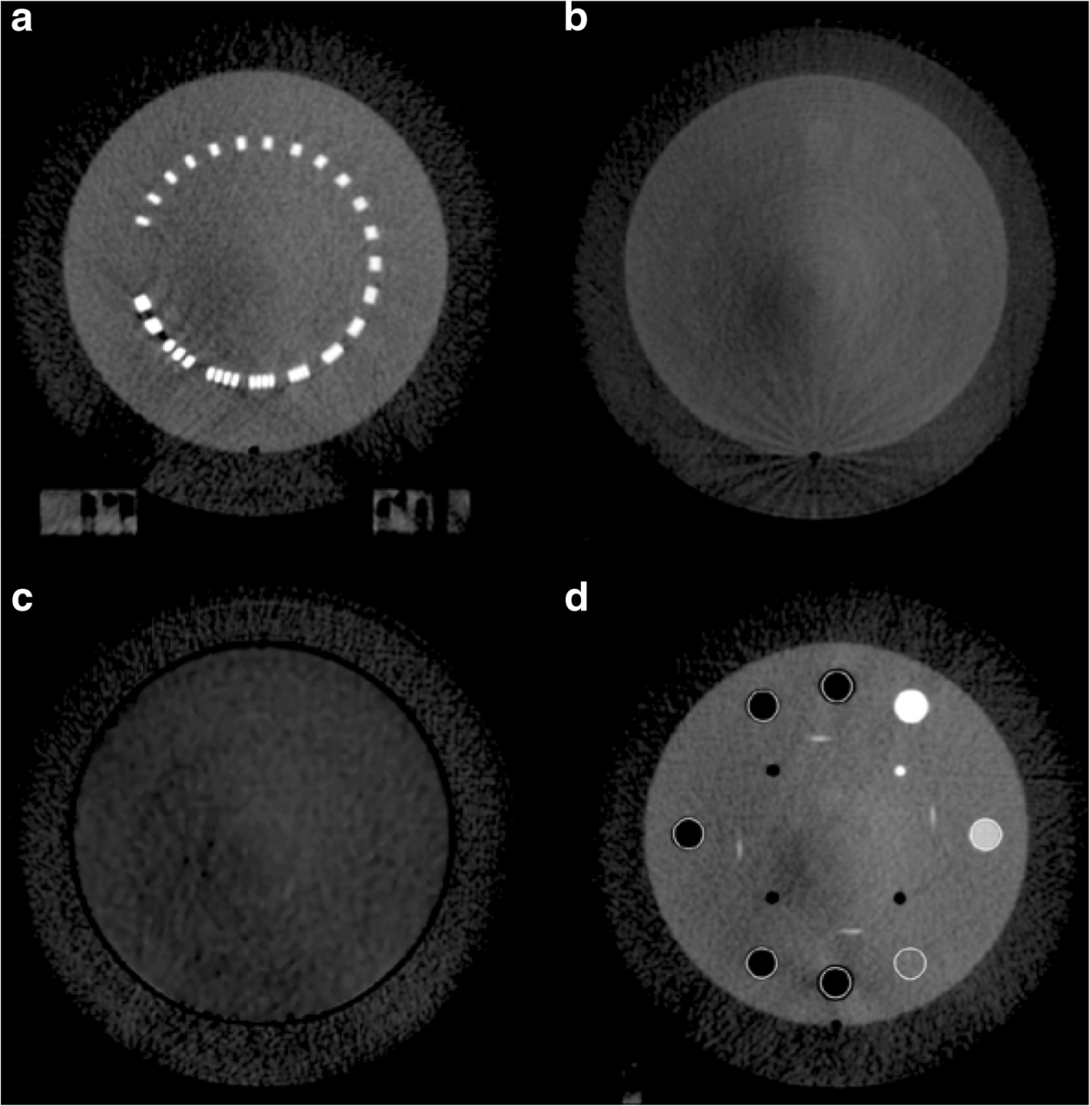
Different slices of the Catphan’s modules used for image quality evaluation. **a** A bar pattern module of increasing spatial frequency (lp/cm), used for determining spatial resolution of the protocols. **b** A low contrast module used in calculating CNR of the various protocols. **c** A homogeneous module used in determining uniformity, with four ROI (anterior, posterior, right, and left) equidistant from a centrally located ROI. **d** A sensitometry module containing materials of known electron density, used in the calculating HU difference from baseline

The following image quality parameters were evaluated: spatial resolution, low contrast detectability, uniformity, and difference in Hounsfield unit (HU) sensitivity from baseline. Image quality metrics were determined for CBCT-ave for all gantry speeds. Modules for image quality evaluation are shown in Fig. 3. All image quality measurements were performed using the Eclipse treatment planning system (Varian Medical Systems, Palo Alto, CA). Certain image quality parameters specified below were normalized to the square root of the mAs, as this represents the anticipated relative noise of the image and provides a kind of benefit-cost ratio as it pertains to image quality.

#### Spatial resolution

A bar pattern module (CTP528) was used to determine the modulation transfer function (MTF) of every protocol to completely characterize the spatial resolution of the imaging system, as seen in Fig. 3a. The spatial frequency *f* of the bar patterns are well known. By calculating the modulation of the bar patterns for various frequencies [24], a raw MTF can be generated via:

3 $$ MT{F}_{raw}(f)=\frac{H{U}_{max}(f)-H{U}_{min}(f)}{H{U}_{max}(f)+H{U}_{min}(f)} $$

These metrics were determined by taking line intensity profiles across the bar patterns. Gaussian fits were applied to the raw data by maximizing the coefficient of determination (R^2^). The final MTF is given by:

4 $$ MTF(f)= \exp \left(-{f}^2/2{\sigma}^2\right) $$

where *σ* represents the fitting parameter. The maximal spatial resolution *f*_*max*_ is defined as the frequency at which the MTF crosses the 10 % level. This was assessed for every protocol using the Gaussian fits. Variation of *f*_*max*_ normalized to the square root of mAs was also evaluated with respect to mAs.

#### Low contrast detectability

Quantification of low contrast detectability can be accomplished via calculation of the contrast-to-noise ratio (CNR). A module containing low contrast cylinders (CTP515) was used to determine CNR, as seen in Fig. 3b. A 1.5 cm in diameter, 1.0 % nominal low contrast cylinder was defined as the region-of-interest (ROI). The CNR is defined as:

5 $$ CNR=\frac{\left|{\overline{x}}_s-{\overline{x}}_{bg}\right|}{\sigma_{bg}} $$

where ${\overline{x}}_s$ represents the mean HU in the ROI, ${\overline{x}}_{bg}$ represents the mean HU of the adjacent background, and *σ*_*bg*_ represents the standard deviation of the background. CNR was calculated for ten individual slices and then averaged producing a single CNR for every protocol. Variation of CNR normalized to the square root of mAs was also evaluated with respect to mAs.

#### Image uniformity

A module composed of homogeneous material (CTP486) was used to determine image uniformity. The mean HU values of four ROI 5.0 cm equidistant from a centrally located ROI were determined, as seen in Fig. 3c. The uniformity index (UI) is given by:

6 $$ UI=H{U}_{max}-H{U}_{min} $$

UI was calculated for ten individual slices and then averaged producing a single UI for every protocol. Variation of UI normalized to the square root of mAs was also evaluated with respect to mAs.

#### HU sensitivity

An imaging system’s ability to accurately characterize a given material’s electron density (ED) is the essence of HU sensitivity. This was accomplished via measurement of various cylinders found in the Catphan’s sensitometry module (CTP404), as seen in Fig. 3d. The mean HU value of each cylinder was measured over ten individual slices and then averaged producing a single mean HU for each cylinder for every protocol. The difference in mean HU from a baseline CBCT scan without motion performed at the same kVp was calculated for every cylinder. Ground truth of a given material’s HU value is represented by the baseline scan [25]. This was used to construct a curve for quantification of HU sensitivity.

### 4D analysis

Retrospective 4D reconstructions were performed maintaining the volume-of-interest while varying reconstruction with 4 uncorrelated phases (CBCT-4 ph) or 10 uncorrelated phases (CBCT-10 ph) as a means to measure differences in reconstruction quality. Reconstruction time increases linearly with the number of projections as well as the number of phases (e.g. approximately 6 and 20 min for CBCT-4 ph and CBCT-10 ph, respectively with 5400 projections). Excursion of this study was 3.0 cm in the SI direction and was calculated by taking the difference in the maximum and minimum position of the air cylinder sensitometry insert. Percent differences between expected excursion and those measured on the 4DCBCT were calculated. To compare excursion between CBCT-4 ph and CBCT-10 ph, root-mean square error (RMSE) for excursion was determined across the different protocols, as projection data for each protocol was independently acquired. For some measured excursion *E* for a given protocol *i*, the RMSE for a given 4D reconstruction is given by:

7 $$ RMSE=\sqrt{\frac{1}{6}{\displaystyle \sum_{i=1}^6}{\left({E}_i-3.0\right)}^2} $$

This was done for both 4D reconstruction techniques.

### Reference dosimetry

**Fig. 4:**
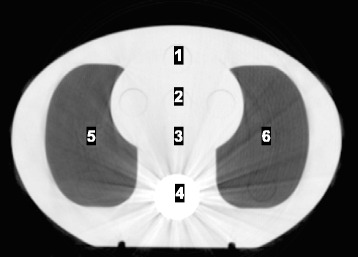
Ion chamber measurement points within the CIRS IMRT Thorax phantom

Following the recommendations of AAPM Task Group 61, an IBA CC13 ionization chamber (IBA Dosimetry GmbH, Schwarzenbruck, Germany), was used to provide absolute dose information [26]. The CC13 was calibrated at an Accredited Dosimetry Calibration Laboratory with a tungsten target, aluminum filtration beam in the kV energy range at 100 and 120 kVp, with reported half-value layers (HVL) of 5.0 and 6.8 mm Al respectively. The HVL for the 125 kVp spectrum of the OBI was determined in air using Gammex high purity aluminum attenuators (Gammex, Middleton, WI). Doses were measured with an electrometer (Keithley 35040 SN 70422) by placing the chamber (IBA CC13 SN 7406) at different coplanar locations within a CIRS IMRT thorax phantom (Model 002LFC, CIRS Inc, Norfolk, VA) placed upon the gating phantom and using the same waveform motion as the Catphan. The plane resided in the central axis at rest. The CIRS is manufactured using proprietary materials intended to mimic the attenuation characteristics of water, bone, and lung within 3.0 % for energies ranging from 50 keV to 25 MeV. The chamber was placed in regions of the CIRS phantom described in Fig. 4a (regions 1–3 for water, region 4 for a bone, and regions 5 and 6 for lung) for every protocol. These measurements were used to determine dose rates in the different tissue mimicking materials.

## Results

### A. Image quality evaluation

**Fig. 5:**
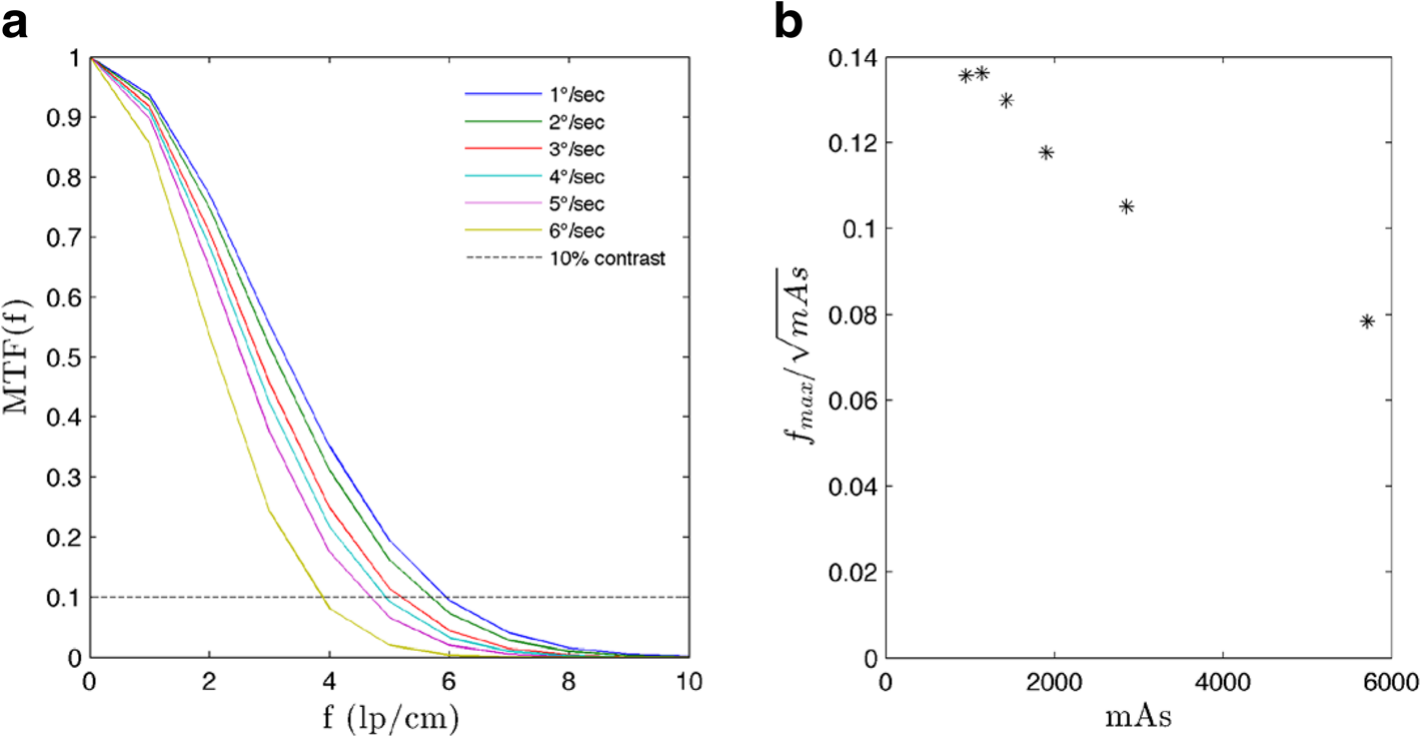
**a** Gaussian fit MTF for CBCT-ave for the various protocols. **b** Maximal spatial resolution normalized to the square root of mAs. As defined above, the maximal spatial resolution corresponds to the spatial frequency at which the MTF crosses the 10 % contrast level

Spatial resolution was evaluated for CBCT-ave. The spatial resolution data are shown in Fig. 5a, exhibiting linearly decreasing behavior with increasing gantry speed. Linearly decreasing maximal spatial resolution with increasing gantry speed is exhibited in Table 2. Evaluation of maximal spatial resolution normalized to the square root of mAs a maximum value at 1140 mAs as seen in Fig. 5b. The inverse square root term in the *f*_*max*_ normalization dominates as mAs increases.

**Table 2:** Maximal spatial resolution (lp/cm) for CBCT-ave at specified gantry speeds

Gantry speed (°/s)					
1.0	2.0	3.0	4.0	5.0	6.0
5.93	5.62	5.14	4.91	4.60	3.82

These spatial frequencies correspond to 10 % contrast of the Gaussian fit

**Fig. 6:**
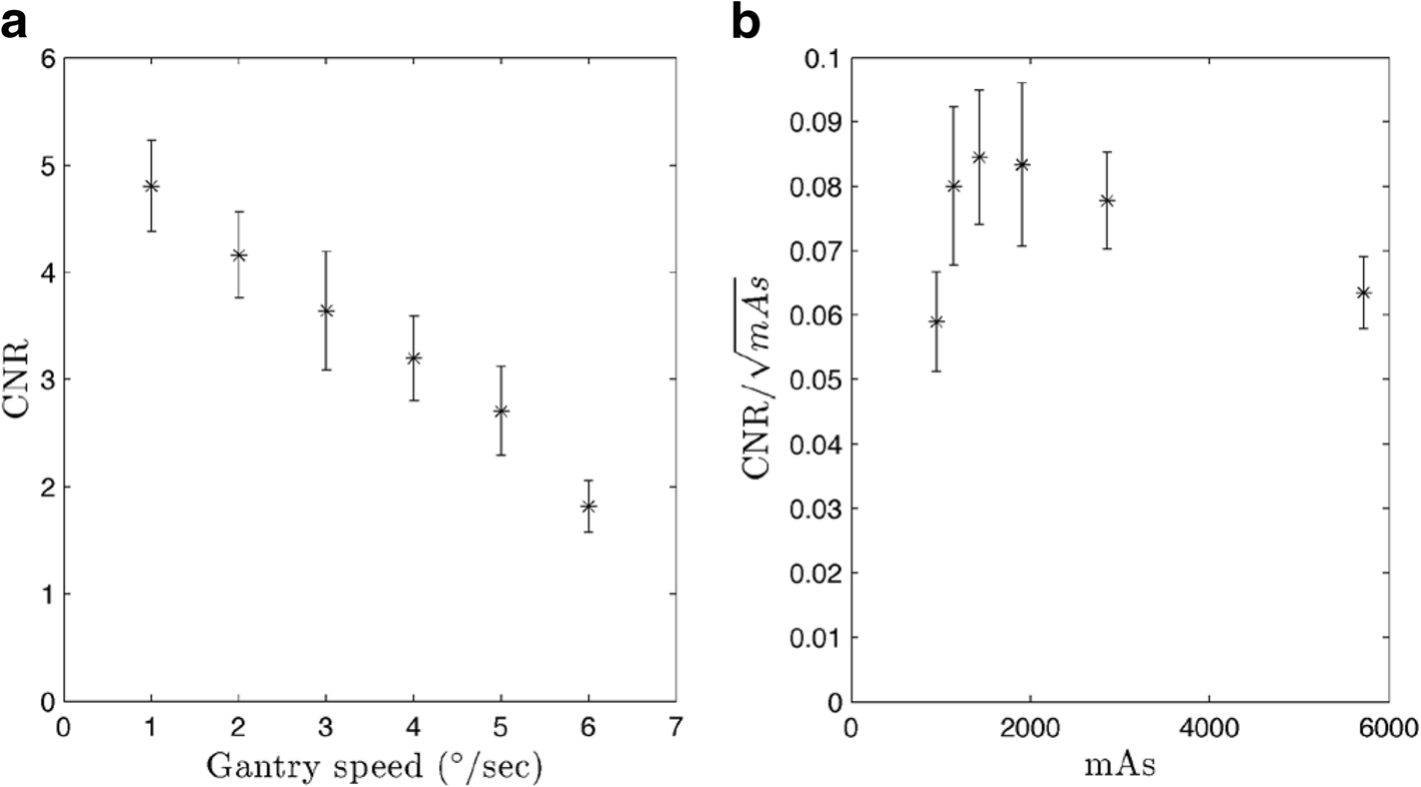
CNR for CBCT-ave. All values were calculated over ten individual slices and then averaged. Error bars represent standard error. **a** CNR exhibits a linear trend as a function of gantry speed. (R^2^ = 0.988). **b** CNR normalized to the square root of mAs. Values were calculated over ten individual slices and then averaged. All error bars represent standard error

**Fig. 7:**
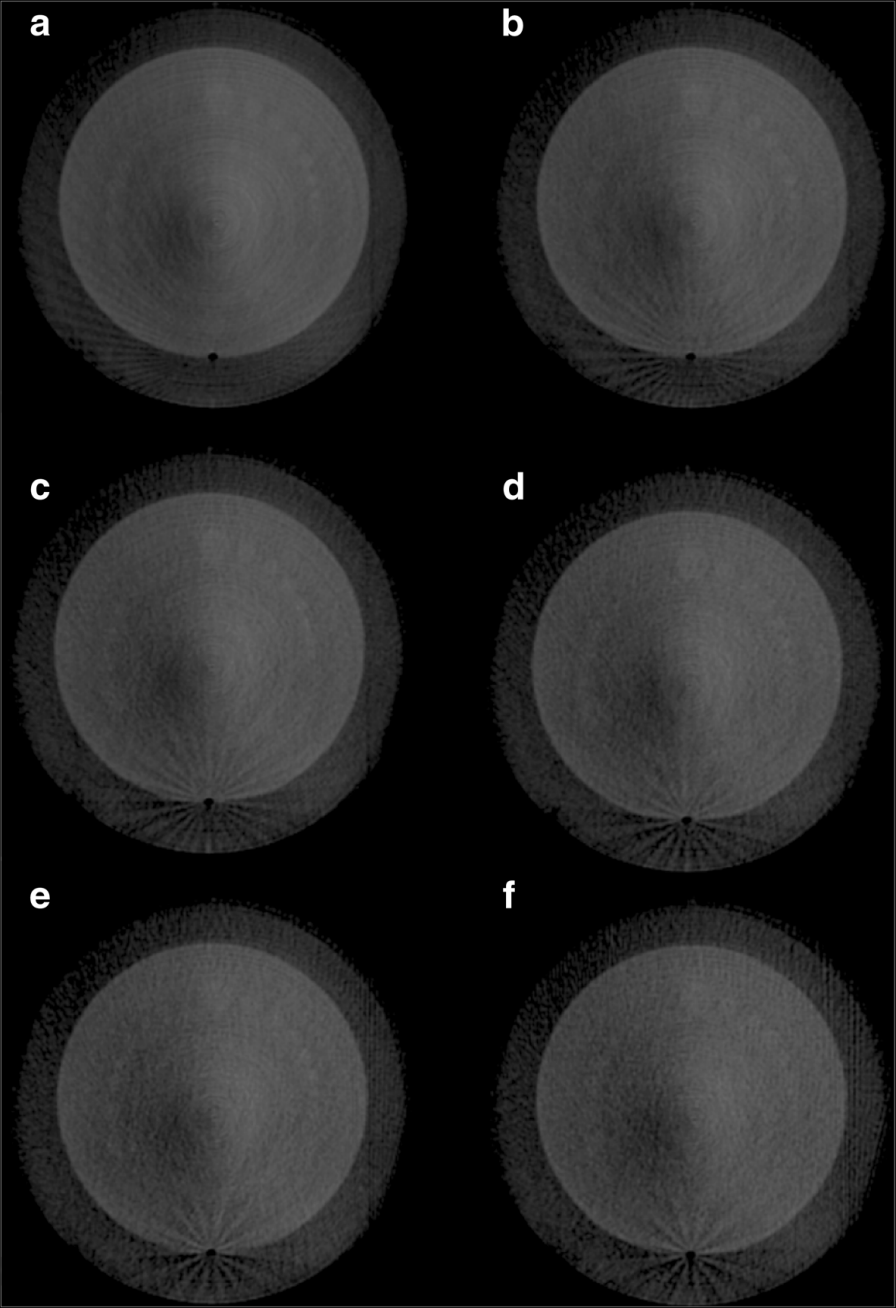
Low contrast module of the Catphan. Images correspond to gantry speeds varying from 1.0 to 6.0°/s for (**a**) through (**f**) respectively

Low contrast detectability exhibited linear decreases, with CNR variations from 4.80 to 1.82 as gantry speed increased from 1.0 to 6.0°/s as seen in Fig. 6a. Evaluation of CNR normalized to the square root of mAs indicates small increases up to 1427 mAs as seen in Fig. 6b. The inverse square root term in the CNR normalization dominates as mAs increases. Qualitative variation of low contrast detectability is exhibited in Fig. 7.

**Fig. 8:**
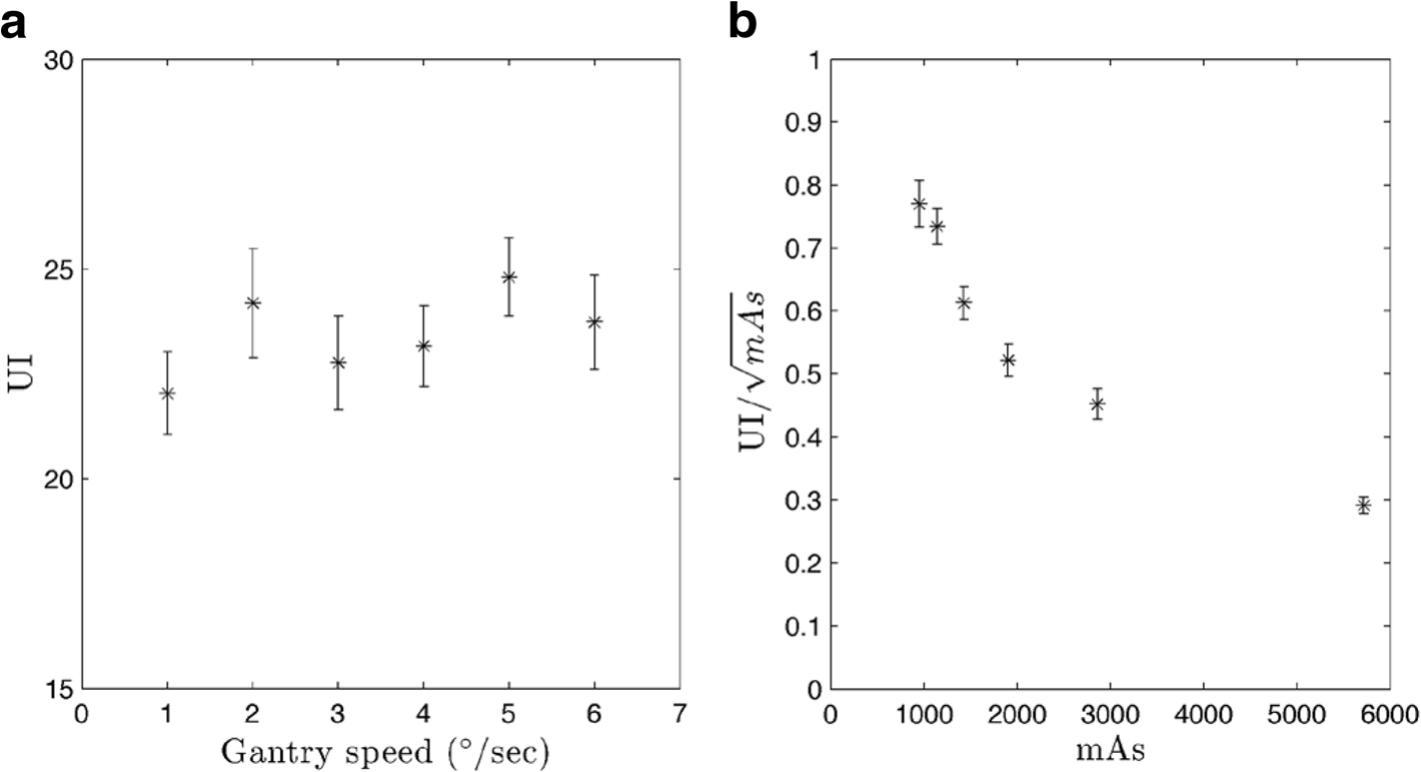
UI for CBCT-ave plotted against **a** gantry speed and **b** normalized to mAs. Values were calculated over ten individual slices and then averaged. All error bars represent standard error

Minimal variations between protocols were observed for image uniformity, as seen in Fig. 8. The difference between the maximum and minimum UI across all the protocols is less than 3.0 HU, as seen in Fig. 8a. Evaluation of UI normalized to the square root of mAs indicates complete dominance of the inverse square root term as seen in Fig. 8b.

**Fig. 9:**
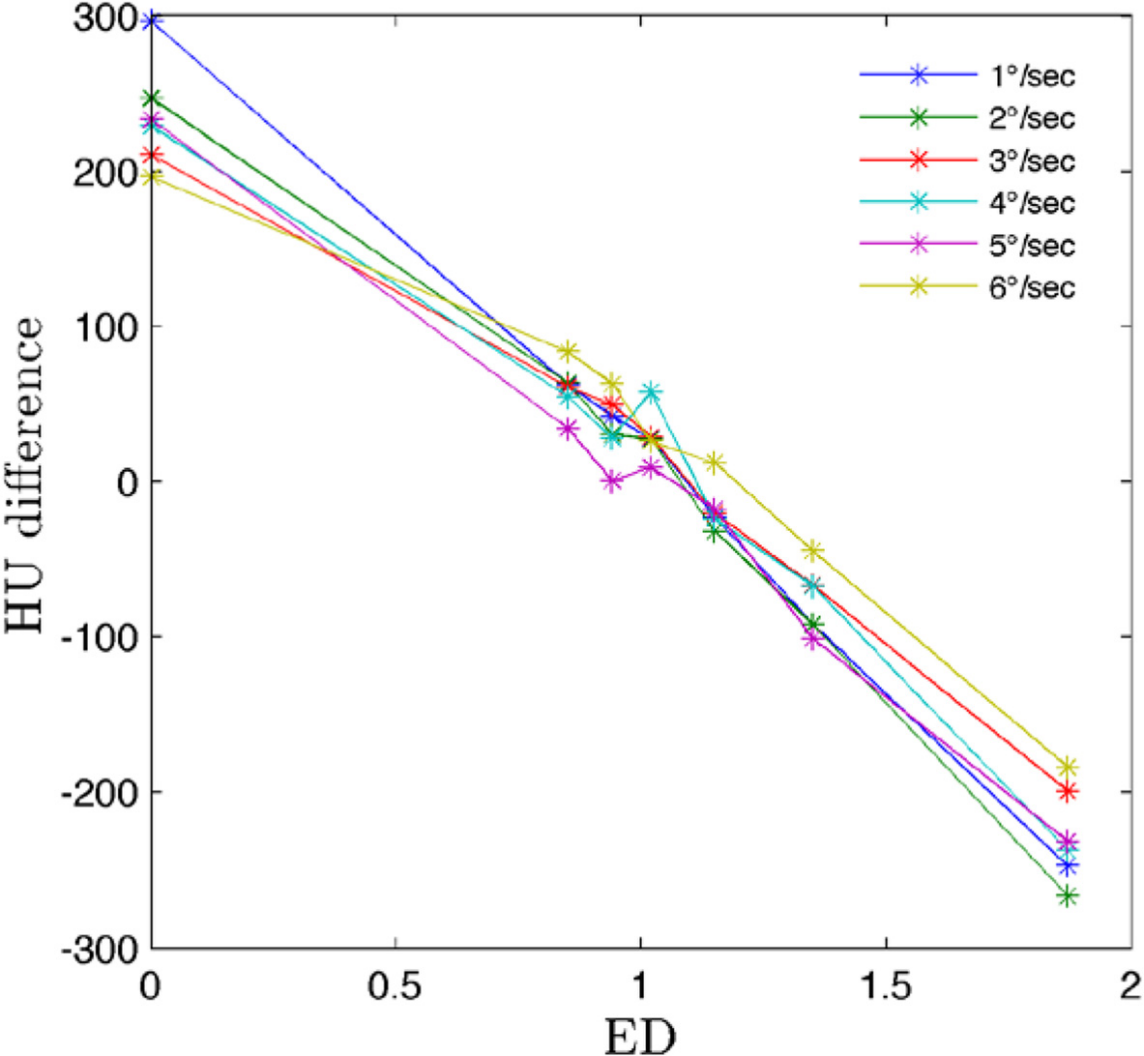
Difference in mean HU from baseline for CBCT-ave. Values were measured over ten individual slices and then averaged

Minimal variations were also observed for HU sensitivity differences from baseline, as seen in Fig. 9. The largest variation in mean HU between protocols was observed for the air cylinder of ED = 0. The difference between the maximum and minimum average HU for air was 100 HU. However, all average HU values are within one standard deviation of each other, in turn making the HU-to-ED curves even more indistinguishable.

Variations in excursion across 4D reconstruction techniques for all protocols were also minimal. The difference between the maximum and minimum measured excursion across all protocols and all reconstruction techniques was less than 1.0 mm. The RMSE in excursion for CBCT-4 ph and CBCT-10 ph were 0.025 and 0.035 cm, respectively. Percent differences in excursion are presented in Table 3. No relationship in error to the known 3.0 cm excursion with the number of projections was observed.

**Table 3:** Percent differences in excursion for the various protocols

	Gantry speed (°/s)						
Volume	1.0	2.0	3.0	4.0	5.0	6.0	RMSE (cm)
CBCT-4 ph	−1.34	1.32	−1.34	0.00	−1.68	0.00	0.025
CBCT-10 ph	1.32	1.00	−0.33	0.00	1.00	0.33	0.035

Positive values represent overestimates while negative values represent underestimates. RMSE across the protocols for a given reconstructed volume is also provided

### Imaging dose evaluation

The HVL measured in air was determined to be 5.02 mm Al. Appropriate mass-energy absorption coefficients were applied to the tissue-mimicking materials [26] Resultant dose rates for a given chamber position are provided in Table 4 below. Comparison to other dosimetry studies is provided in the discussion below.

**Table 4:** Dose rates (E-3 cGy/mAs) measured in different tissue-mimicking materials

Chamber position					
1	2	3	4	5	6
2.86	3.03	2.84	5.18	2.49	2.30

## Discussion

CBCT finds great utility in radiation oncology. CBCT image acquisition prior to delivery of radiation can be registered to previously acquired CT scans used for treatment planning and provides images for retrospective dose accumulation studies. However, standard 3D acquisition and reconstruction does not allow visualization of mobile lesions as a function of respiratory phase and is susceptible to motion artifacts such as blurring and distortion. Moreover, average 3D reconstructions may result in underestimates in excursion due to short periods of time spent at peak inhalation/exhalation. The use of 4DCBCT has the potential to mitigate these issues by assigning projections to specific breathing cycle phases. This is feasible due to changes in the diaphragm, causing organ motion with the diaphragm’s dynamics. This in turn justifies the use of surrogate signals such as skin surface mapping or infrared markers, as the position of the surrogate is variable with respect to time [4, 7].

A major difficulty in 4DCBCT is the limited number of projections per phase bin. Insufficient projection data leads to aliasing artifacts. Li and Xing proposed slow gantry rotation (SGR) and multiple gantry rotation (MGR) acquisition techniques to increase the number of projections for a given phase [27]. They showed SGR produced superior images to MGR for the same mAs when compared to 3DCBCT counterparts.

Time-averaged 4DCBCT images over the respiratory cycle are used for patient localization compared against time-averaged 4DCT images [28]. The image quality of 4DCBCT images plays an important role in 4D dose accumulation. Anatomical voxels are mapped from the 4DCBCT at treatment position to a reference 4DCT. However, the low number of projections per phase in the 4DCBCT image may result in degraded image quality, specifically streaking at high contrast boundaries and blurring, in turn leading to unrealistic dose calculations [29].

**Fig. 10:**
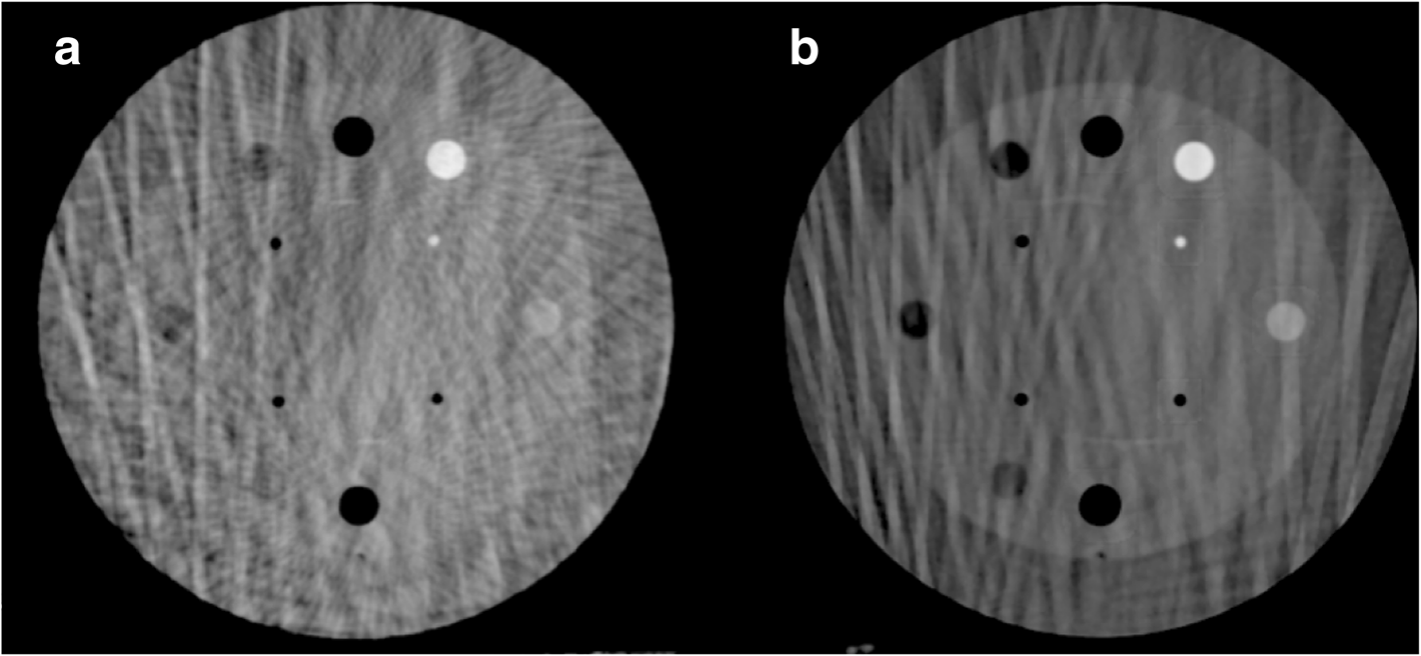
Streaking artifacts present in the sensitometry module for the same CBCT slice and the same phase. These slices correspond to **a** CBCT-4 ph and **b** CBCT-10 ph for a gantry speed of 1°/s

In this study, contrast-based image quality metrics were affected by variable gantry speed. Yoganathan et al. have shown similar results in their analysis of an Elekta 4DCBCT system using a Catphan® 600 [30]. Specifically, spatial resolution and low contrast detectability decreased with increasing gantry speed and a fixed frame rate. Yoganathan et al. further demonstrated that reductions in 4DCBCT image quality parameters may result in underestimates of target volumes when compared to 4DCT contoured volumes used in treatment planning. Improved metrics with slower acquisition leading to increased projections/mAs is likely due to decreases in noise. Saturation of these metrics occurs when viewing the parameters against the number of projections/mAs. This is related to the imaging system being unable to decrease noise levels further. However, normalization of image quality parameters by the square root of mAs indicates marginal improvements in maximal spatial resolution and low contrast detectability passed a given mAs and a lack of improvement with respect to uniformity. The presence of artifacts like streaking, as seen in Fig. 10, makes evaluation of image quality for 4D volumes difficult. Cooper et al. have described streaking and star artifacts in 4DCBCT being attributed to the presence of two sampling frequencies – a higher sampling frequency for the projections in a given phase bin and a lower sampling frequency between phase bins [31]. In implementing a FDK cone-beam reconstruction with so few projections per phase, materials with stronger attenuation characteristics are not balanced out in the back projection process in turn leading to star and streaking artifacts [18].

HU sensitivity and excursion did not exhibit noteworthy differences in CBCT-ave with variable gantry speed, especially when accounting for error in the case HU sensitivity. With respect to excursion, across all the protocols, percent differences were less than 2.0 %. In comparing 4 phase against 10 phase reconstructions, both RMSE are less than half a millimeter. This indicates that the 4D reconstructions reproduce realistic excursion. In this particular study, the 15 fps acquisition and characteristics of the waveform motion mitigate issues of latency. Based on the motion waveform used, acquisition may miss 0.8 mm of motion between frames on average. For more irregular breathing patterns, this may not be the case resulting in errors in the phase sorting process.

Characterization of image quality for given tube current settings (mAs) for 4DCBCT has been previously performed for systems including the Varian’s Acuity™ simulator and Trilogy™ OBI (Varian Medical Systems, Palo Alto, CA) [25, 29]. These studies focused primarily on a comparison of image quality relative to some reference using SGR or MGR acquisition techniques. Dose was characterized via mAs, as dose is directly proportional to mAs [29].

The CTDI_100_ metric is not appropriate for dosimetric evaluation of CBCT scans due to its inability to accommodate and record the whole primary beam and scattered radiation [32]. Hence 4DCBCT was evaluated dosimetrically on an absolute scale. Ion chamber measurements from this study produced dose rates of 3.0 × 10^−03^ cGy/mAs at isocenter in a water equivalent medium. Li et al. reported for the Varian Acuity and Trilogy systems, a 125 kVp spectrum and tube current of 80 mA produced dose rates at isocenter of 2.92 × 10^−03^ and 2.79 × 10^−03^ cGy/mAs, respectively [33]. Similarly, Gardner et al. reported isocenter doses using film in a Wellhofer phantom of (2.72 ± 0.11) × 10^−03^ cGy/mAs using TrueBeam OBI and 125 kVp setting. McMillan et al. compared measured dose rates to dose rates determined using Monte Carlo methods for the OBI kV imaging system integrated into the Varian Novalis Tx radiosurgery platform (Varian Medical Systems, Palo Alto, CA) [34]. A dose rate of 2.23 × 10^−03^ cGy/mAs at isocenter was calculated for a comparable pelvic CBCT protocol using a 125 kVp spectrum and a 32 cm diameter cylindrical CTDI phantom composed of homogeneous polymethyl methacrylate. Variation in dose rate in this study from others is attributed to spectral differences and variations in phantom composition and geometry.

While increasing the cumulative mAs increases the quality of spatial resolution and low contrast detectability, the cost of linearly increasing dose and increased 4D image acquisition and reconstruction time must be considered. Artifacts such as streaking in 4DCBCT are largely attributed to the lack of projection data being used for reconstruction. In this particular study, the incremental improvements in image quality above 2000 projections become unjustified compared to the linear increases in dose. This corresponds to a gantry speed between 2 and 3°/s. Clinical use of 4DCBCT systems for patients relies entirely on intended application.

## Conclusions

A quantitative analysis of the Varian OBI’s 4DCBCT capabilities was explored for making clinical decisions. The effects of gantry speed on 4DCBCT image quality and dose have been investigated yielding anticipated results. When using the same technique settings (kVp), variations in gantry speed change the number of projections used for reconstruction but maintain the mAs per projection. Unsurprisingly, contrast-based image quality metrics were found to decrease linearly with increasing gantry speed but show marginal improvements when appropriately normalized to the square root of mAs. The benefit of increased contrast comes at the cost of increased dose, slower acquisition time, and longer 4D reconstruction time; the clinical benefits of improved image quality must be weighed against the costs of decreased efficiency and increased imaging dose. The future of 4DCBCT’s clinical utility relies on further investigation of image optimization, requiring more than just large-scale increases in mAs to improve image quality.

## Abbreviations

4DCT, four-dimensional CT; AP, anterior-posterior; CBCT, cone-beam CT; CBCT-10 ph, ten phase 4DCBCT reconstruction; CBCT-4 ph, four phase 4DCBCT reconstruction; CBCT-ave, average CBCT reconstruction; CNR, contrast-to-noise ratio; ED, electron density; FDK, Feldkamp-Davis-Kress; fps, frames per second; HU, Hounsfield unit; HVL, half-value layer; MGR, multiple gantry rotation; MTF, modulation transfer function; NSCLC, non-small cell lung cancer; OBI, On-Board Imager®; RMSE, root-mean square error; ROI, region-of-interest; RPM, real-time position management; SBRT, stereotactic body radiotherapy; SGR, single gantry rotation; SI, superior-inferior; UI, uniformity index
